# Vascularization in mTOR Mouse Mutants: An Effort Not in Vein

**DOI:** 10.1523/ENEURO.0122-23.2023

**Published:** 2023-07-19

**Authors:** Soad Elziny, Peter B. Crino

**Affiliations:** 1 Program in Neuroscience, University of Maryland School of Medicine, Baltimore, 21201 MD; 2 Department of Neurology, University of Maryland School of Medicine, Baltimore, 21201 MD

## Abstract

**Highlighted Research Paper:** M. Dusing, C. L. LaSarge, A. White, L. G. Jerow, C. Gross and S. C. Danzer, “Neurovascular Development in *Pten* and *Tsc2* Mouse Mutants.”

The mechanistic target of rapamycin (mTOR) signaling pathway is activated by cellular inputs from growth factors, ATP, amino acids, and oxygen levels. Mutations in mTOR pathway genes (MPG) such as *TSC2* and *PTEN*, both negative regulators of mTOR, are linked to developmental brain malformations, intellectual disability, autism, and epilepsy (Mirzaa et al., 2016; Marsan and Baulac, 2018). *TSC2* and *PTEN* mutations lead to mTOR pathway hyperactivation during fetal brain development causing morphologic and physiological abnormalities in the developing brain, i.e., neuronal hypertrophy, aberrant dendritic architecture, altered cortical lamination, brain overgrowth, and network hyperexcitability. Curiously, the effects of MPG variants on brain vascular development have not been deeply investigated although the vascular network results from the concomitant development of both neural and vascular components via angiogenesis (generation of new blood vessels from preexisting ones) and vasculogenesis (generation of blood vessels *de novo*) starting early in embryonic development. Thus, it seems likely that MPG might have effects on blood vessel structure and function. For example, MPG variants causing mTOR activation may alter expression of vascular remodeling genes such as *VEGF*, *Ang-1/2*, *HIF-1α*, and matrix metalloproteinases (Xue et al., 2018; Broekaart et al., 2020) and indeed, vascular remodeling has been reported in heterozygous *Tsc2* Eker rats (Kútna et al., 2020). Seizures associated with MPG variants may disrupt the blood-brain-barrier integrity (Mendes et al., 2019).

Recently, Dusing et al. (2023) have investigated the effects of mTOR hyperactivation on brain vasculature because of loss of either *Pten* or *Tsc2*, in three mouse models comparing a focal *Pten* knock-out (KO) in dentate granule cells (*Gli1-DGC-P10* KO) following tamoxifen treatment at postnatal day 14 (P14), a conditional *Pten* knock-out in forebrain excitatory neurons (*CamK2α-FB-P10* KO), and an AAV9-mediated *Tsc2* knock-out in cortical excitatory neurons (targeted injection of AAV9-CaMK2αshort>mCherry:T2A:Cre:WPRE vector; f-*Tsc2* KO). Blood vessel area was significantly increased (51.9%) in *DGC-P10* KO mice at 10 weeks of age relative to both earlier timepoints. To determine whether the observed increase in vessel area also produced an increase in vessel density, dentate volume for each brain slice was examined. Quantification of dentate vessel volume revealed significant increases across groups with *DGC-P10* KO vessel volume greater at 10 weeks (51.8%) compared with previous timepoints and controls. However, when dentate vessel area was normalized to dentate volume (to calculate vessel density), no significant differences were seen across age groups. Using confocal image stacks and Neurolucida 360 software analysis, the authors were able to create 3-D-reconstructed images of vasculature in each dentate, and gain in-depth quantitative analysis of vessel length, volume, diameter, and tortuosity (how “twisted” a vessel is). They noted significantly increased vessel length and volume in 10-week *DGC-P10* KO mice; however, when normalized for dentate volume increase (because of *Pten* KO), there was no significant difference in vessel density. Vessel diameter and tortuosity were also not different among groups. It appeared that increasing vessel area was driven by lengthening, with no marked changes in diameter or vessel path. In parallel experiments, blood-brain barrier leakage was assessed in the *DGC-P10* KO group using AF488-conjugated bovine serum albumin before perfusion but showed no statistically significant alterations (Fig. 4).

The investigators hypothesized that the lack of change in vascular density was because of the comparatively smaller population of affected neurons in the *DGC-P10* KO group, thus, they next assayed the *FB-P10* KO mice where vessel area in cortex and hippocampi was larger than controls, however, when normalized to brain volume, there was again, no significant changes in vessel density. Two mice expressed unexpected *germline* loss of one *Pten* allele (vs conditional), and those values seemed to overlap with the values of *FB-P10* KO group, indicating that *Pten* heterozygosity does not change the phenotype and that even loss of *Pten* from neurons is not enough to drive persistent hypervascularization. In parallel experiments, the expression of the angiogenic factor VegfA was increased significantly (65%) in the *FB-P10* KO mice relative to controls.

Finally, in the f-*Tsc2* KO strain, there was increased vascularity in areas of focal *Tsc2* loss. *Post hoc* analyses confirmed that there was significantly increased vascular density in the mCherry-expressing hemisphere relative to the nonexpressing hemisphere within f-*Tsc2* KO strains compared with controls. This is contrary to the effect seen in the previous FB-P10 KO group, suggesting that *Pten* and *Tsc2* KOs create differing vascular characteristics.

Overall, Dusing et al. (2023) performed a thorough and rigorous analysis. Indeed, by using varying analysis techniques (3-D reconstructive analysis vs 2-D analysis), defined regional expression of *Pten* deletion (DGC vs FB), and distinct mTOR regulators (*Tsc2* vs *Pten*), the group provided solid evidence that mTOR hyperactivation results in distinct profiles of altered vascularization depending on genotype and brain region. In *PTEN*-associated hemimegalencephaly or megalencephaly and in TSC, altered vascular structure could have important implications for understanding disease pathogenesis or treatment and indeed, there is an increase in microvasculature in white matter of TSC cortical tubers (Veersema et al., 2019). Vascular anomalies are identified in patients with *PTEN* germline mutation syndromes (Tan et al., 2007). The significance of the current findings to patients with either *PTEN* or *TSC2* mutation syndromes should be defined in further studies.

There are some limitations to the study. Focal loss of either *PTEN* or *TSC2* is uncommon in the hippocampus in human patients and thus analysis of the hippocampus may not yield insights into human disease. The choice of a P14 tamoxifen induction to *Pten* KO does not model when *PTEN* mutations usually exert an effect, i.e., during embryonic development. Since all three mouse model platforms target a neuron-specific KO, consideration of how noncell autonomous mechanisms might alter brain vasculature would be interesting. It would have been helpful to discuss how loss of either *Pten* or *Tsc2* in neurons might lead to altered vascular structure, an effect that likely requires signaling to endothelial cells. The *DGC-P10* KO also hits small numbers of astrocytes (∼1%) in cortex and midbrain but not endothelial cells. Assay of VegfA levels in the *DGC-P10* KO mice or f-*Tsc2* KO strains would have been intriguing since Vegf levels are elevated in human TSC and might shed mechanistic light into how loss of *Pten* or *Tsc2* might alter vasculature in various brain regions. Further work could aim to reconcile the elevation in VegfA, a known angiogenesis factor, with the paucity of overall changes in vascularization in either *Pten* or *Tsc2* KO.

## Synthesis

Reviewing Editor: William Stacey, University of Michigan
